# Elevated Anion Gap Metabolic Acidosis With High Osmolar Gap and Increased Serum Acetone Level: A Case Report

**DOI:** 10.7759/cureus.27085

**Published:** 2022-07-20

**Authors:** Debargha Basuli, Sasmit Roy

**Affiliations:** 1 Internal Medicine, East Carolina University, Greenville, USA; 2 Nephrology, University of Virginia, Lynchburg, USA; 3 Nephrology, Liberty University Medical School, Lynchburg, USA

**Keywords:** acetone, alcohol toxicity, osmolar gap, anion gap acidosis, acetone toxicity

## Abstract

Acetone poisoning, although not very common, can present with varied signs and symptoms. High acetone levels in serum can be due to exogenous exposure or endogenous production of acetone. Unlike certain alcohol toxicities, acetone does not cause high anion gap metabolic acidosis.

A 69-year-old male presented to our service with shock and acute encephalopathy and required intensive care support. Initial laboratory investigation showed high anion gap metabolic acidosis with high osmolar gap. Serum acetone level was elevated. Clinicians need to be aware of how to elucidate such metabolic disturbances and associated toxicities.

## Introduction

High anion gap metabolic acidosis is mainly caused by an overdose of glycols, 5-oxoproline/pyroglutamic acid, L-lactate, D-lactate, methanol, aspirin, renal failure, and ketoacidosis [1]. High serum osmolarity with an osmolar gap can be yielded by hyperlipidemia or hyperproteinemia, sugars (mannitol, sorbitol) [2], and alcohols. High anion gap and high osmolarity with an osmolar gap are two different entities but can co-exist in some instances and can be an essential clue for certain toxic alcohol exposure.

Acetone, by itself, does not cause high anion gap metabolic acidosis but can present a complex clinical picture with associated metabolic acidosis due to other co-existent conditions. Although much less common than alcohol poisoning, there are reported cases of acetone toxicity in humans. The etiology of elevated acetone can be multifold and may not always be due to direct ingestion. Here we present a case presenting with acute encephalopathy and shock, with high anion gap acidosis and an osmolar gap with elevated acetone level in serum with a didactic discussion about how to interpret such findings.

## Case presentation

A 69-year-old African American male was found unresponsive in his bathtub. He was taken to the local emergency room, where he remained obtunded. Upon presentation to the emergency room, the patient's blood pressure was 79/56 mm Hg, and his heart rate was 121 beats per minute. EKG showed new-onset atrial fibrillation with a rapid ventricular response. He was hypothermic with a rectal temperature of 90.7 °F. Initial laboratory results showed elevated WBC count, low bicarbonate of 7 mEq/L, elevated creatinine of 2.58 mg/dL (baseline creatinine was 1 mg/dL), and a lactic acid level of 1.3 mmol/L. Arterial blood gas showed a pH of 7.21, and partial pressure of carbon dioxide (PCO2) of 22 mm Hg. The anion gap was elevated at 30. Serum osmolality was 362 mOsm/kg, and calculated osmolality was 346 mOsm/kg, with an elevated osmolar gap of 16 mOsm/kg. Urine was positive for trace ketones. Other laboratory values are shown in Table 1. CT scan of the head showed no acute intracranial pathology. His blood pressure did not improve with multiple intravenous fluid boluses, and therefore was started on norepinephrine, vasopressin, and empirical antibiotics. The patient was intubated for airway protection and was admitted to the intensive care unit (ICU) for further management. In the ICU, he was maintained on vasopressor support and mechanical ventilation. Stress dose of steroid with hydrocortisone of 50 mg every six hours was started. Given the elevated anion gap and osmolar gap, acute alcohol toxicity was suspected, and he was given an empirical dose of fomepizole (15mg/kg). The urine toxicology panel came back negative. However the volatile screen in blood was positive for high level of acetone (50 mg/dL) and negative for isopropyl alcohol, methanol, and ethanol (Table 1). A chest X-ray showed clear lungs. The patient was started on continuous renal replacement therapy (CRRT) for acute kidney injury with severe acidosis. After 48 hours on CRRT, the anion gap and the osmolar gap closed and the acetone level decreased. Patient’s clinical status improved and he was extubated two days after admission to the ICU.

**Table 1:** Patient's laboratory values on presentation. pCO2: partial pressure of carbon dioxide, BUN: blood urea nitrogen, pO2: partial pressure of oxygen

	Patient's Laboratory Values	Reference Range
Sodium	149	136 - 145 mEq/L
Potassium	3.9	3.5 - 4.5 mEq/L
Chloride	112	98 - 107 mEq/L
Bicarbonate (TCO2)	7	23 - 31 mEq/L
Anion Gap (calc.)	30	4 - 12 mEq/L
Glucose	138	70 - 105 mg/dL
BUN	113	8 - 26 mg/dL
Creatinine	2.58	0.72 - 1.25 mg/dL
Osmolality (calc)	346	
Osmolality, serum	362	270 - 295 mOsm/kg
Lactic Acid	1.3	0.5 - 2.0 mmol/L
pCO2, arterial	22	35 - 45 mm Hg
pH, arterial	7.21	7.35 - 7.45
pO2, arterial	117	85 - 95 mm Hg
Valproic Acid	<12.5	50.0 - 100.0 ug/mL
Acetaminophen	<2.0	10.0 - 30.0 ug/mL
Salicylates	<2.0	2.8 - 20.0 mg/dL
Ethanol	<10	<10 mg/dL
Isopropanol	<5	<5 mg/dL
Methanol	<20	<20 mg/dL
Acetone	50	<5 mg/dL
pH, urine	5	5.0 - 8.0
Protein, urine	Negative	Negative
Glucose, urine	Negative	Negative
Ketones, urine	Trace	Negative

He was transferred then to the non-ICU ward and discharged after three days on his home medications. The patient had a past medical history of schizophrenia and psychosis and was on risperidone and valproic acid. He denied any accidental or intentional ingestion of any chemical before the presentation. 

## Discussion

To understand and interpret the laboratory findings of this case, a brief discussion about causes of high anion gap metabolic acidosis with high osmolar gap is warranted. Increased anion gap is caused mainly by glycols, 5-oxoproline/pyroglutamic acid, L-lactate, D-lactate, methanol, aspirin, renal failure, and ketoacidosis [1]. Osmolar gap is defined as the difference between actual serum osmolarity and calculated serum osmolarity. Increased osmolar gap (defined as >10 mOsm/g [3]) is caused by hyperlipidemia or hyperproteinemia [2], sugars (mannitol, sorbitol), and alcohols. Increased osmolar gap is clinically used to screen for toxic alcohol ingestion such as ethanol or certain toxic alcohols, including methanol, ethylene glycol, diethylene glycol, propylene glycol, and isopropanol. Among these alcohols, isopropanol does not cause metabolic acidosis [4]. Ethanol is the most common clinical scenario when both anion and osmolar gaps are elevated. This was ruled out in our case as the blood alcohol level was negative. A volatile screen was negative for isopropanol and methanol. Ethylene glycol was a consideration for high anion metabolic acidosis with an osmolar gap in the patient as this can also result in renal failure (which the patient had) but the volatile screen did not report ethylene glycol concentration. Unfortunately, some laboratories require a specific order for ethylene glycol when ordering toxic alcohol volatile screens. 

Valproic acid (VPA) toxicity can manifest with acidosis, shock, and multi-organ failure with encephalopathy [5] like our patient. VPA being a weak organic acid can generate an anion gap acidosis [6]. We tested for VPA in the patient and it was negative. Elevated serum anion and osmolar gap have been found in lactic acidosis in the absence of alcohol exposure and alcoholic ketoacidosis [7]. Our patient’s lactic acid was not elevated, but urine was positive for trace ketone bodies.

Interestingly, the volatile screen of the patient was positive for acetone. Acetone, being a non-acid ketone body does not generate an anion gap acidosis by itself but may cause an elevated anion gap by ketosis, or uremia from kidney injury. Acetone metabolites like acetol and 1,2-propanediol can theoretically cause elevated osmolar gap but have been rarely reported [8]. A positive acetone level in serum or urine in absence of any alcohol is possible and has been reported in the literature [1]. The etiology of elevated acetone can be multifold and is generally not due to direct ingestion of acetone itself like this patient who denied any acetone ingestion. Other sources include isopropyl alcohol metabolism and endogenous generation. Isopropanol is converted into acetone by oxidation with hepatic alcohol dehydrogenase. Since the half-life of isopropanol (1-3 hours) is much shorter than acetone (17-27 hours) [1], patients with isopropanol ingestion may be positive for acetone but negative for isopropanol at the time of presentation. Acetone is endogenously produced during states of ketosis/ketoacidosis (diabetic, starvation, or alcoholic) through lipid oxidation. It is possible that this patient had severe ketoacidosis going on. Although his urine was positive for trace ketones only (beta-hydroxybutyrate was not checked), urinalysis measures acetoacetate and in certain ketoacid processes, there is a metabolic shift towards beta-hydroxybutyrate generation with minimal acetoacetate present. 

Acetone toxicity by ingestion or other exposure has been reported in the literature. Acetone is a relatively nontoxic compound. Acetone poisoning is uncommon in human, and serious life-threatening complication is even rarer. At toxic levels, it presents with common symptoms of nausea and vomiting, drowsiness, abdominal pain, and fruity odor. More severe symptoms include stupor and coma [9]. A dose of 50 mL (40 g) or more is found to be toxic in humans, and the minimum lethal dose for an adult man is estimated to be 100 mL (80 g) [10]. 

Acetone is highly soluble in water, making it highly bioavailable [11]. Most of the elimination of acetone occurs mainly through the lungs, with a variable half-life of over 10 hours [12]. Acetone is metabolized into glucose and patients can present with hyperglycemia like the patient in our case [13]. 

Acetone poisoning occurs mainly through ingestion, although routes of entry like inhalation and absorption through skin are also reported. Table 2 shows a summary of case reports of acetone toxicity with the information on the route of entry, and the presenting symptoms and findings. Toxicity from acetone can affect any system. Although hypotension has been reported in acetone poisoning earlier [14], there is only one case reported recently where a patient presented with shock after being resuscitated from asystole [15]. Our patient presented with hypotensive shock, requiring care in ICU and vasopressor support. It was unknown if the shock in our patient was a direct result of acetone toxicity, or a result of any volume loss through the gastrointestinal tract before presenting to the hospital. Other causes of shock were ruled out, including an infectious etiology. An echocardiogram showed a normal ejection fraction without any signs of cardiomyopathy. 

**Table 2:** Summary of case reports on acetone toxicity available in literature.

Case Study Reference	Route of exposure	Presenting Symptoms and findings
Renshaw et al, 1956 [16]	Absorption from lightweight cast	Unconsciousness, vomiting, hematemesis
Hift et al, 1961 [17]	Absorption from synthetic plaster case	Drowsiness, irritability, abdominal pain, coffee-ground emesis, acetone breath
Gitelson et al, 1966 [13]	Ingestion	Coma
Savage et al, 2007 [18]	Ingestion (9-month-old baby sucking on nail polish remover pads)	Coma
Kumarvel et al. 2007 [19]	Ingestion	Respiratory distress, tachycardia, hypertension, tachypnea, fruity odor on breath
Piatkowski et al, 2007 [20]	Inhalation	Skin burns from acetone, corneal erosion, bronchial injury
Kallenberg et al. 2008 [21]	Possible ingestion	Lethargic state with markedly increased muscle tone in the upper extremities. MRI brain showed vasogenic edema
Umeh et al, 2021 [15]	Ingestion	Cardiac arrest/asystole, hypotension, altered mental status, ulcerated and necrotic esophagus and stomach on endoscopy, ultimately death

Management of acetone toxicity remains conservative support. Patients presenting toxic effects of acetone should be closely observed on cardiac monitor and pulse oximetry. Endotracheal intubation may be considered for airway protection when indicated. Intravenous fluid resuscitation may be required to correct any hypotension. Vasopressors may occasionally be required as seen in our case. Although in vitro studies have shown that activated charcoal helps with the adsorption of acetone [22], there are no reported cases of an attempt of gastrointestinal decontamination with activated charcoal. 

## Conclusions

In summary, we presented a case of elevated anion metabolic acidosis with an osmolar gap in a patient presenting with acute encephalopathy and shock and a discussion about the differential etiologies that would give such a clinical picture. Increased acetone levels can be found without any other toxic alcohols, after acetone or isopropanol exposure or from endogenous processes. Severe symptoms from acetone poisoning are rare, although possible, and may require intensive care. 
